# Functional Reconstruction of Lower Eyelid Using Paramedian Forehead Flap Combined with Frontalis Muscle and Periosteum

**DOI:** 10.1055/a-2521-2337

**Published:** 2025-05-15

**Authors:** Riku Katayama, Takako Fujii, Chie Kanayama, Hisashi Sakuma

**Affiliations:** 1 Department of Plastic and Reconstructive Surgery, Shizuoka Cancer Center, Shizuoka, Japan; 2 Department of Plastic and Reconstructive Surgery, Tokyo Dental College Ichikawa General Hospital, Ichikawa, Chiba, Japan; 3 Department of Plastic and Reconstructive Surgery, Tokyo Metropolitan Children's Medical Center, Tokyo, Japan

**Keywords:** forehead flap, lower eyelid, neurotization

## Abstract

Both cosmetic and functional aspects are important in reconstructing the lower eyelid tissue defects. In this case report, we describe a two-stage reconstruction of a skin defect, including the orbicularis oculi muscle, after resection of a basal cell carcinoma using a paramedian forehead flap combined with the frontalis muscle and periosteum. In the first stage, the paramedian forehead flap, including the frontalis muscle and periosteum, was elevated, the periosteal flap was fixed to the outer orbital periosteum to lift the lower eyelid, and the skin flap, including the frontalis muscle, was sutured to the defect. In the second stage, the flap was divided and the frontalis muscle flap was sutured to the medial palpebral ligament. Electromyography at 1 year postoperatively confirmed neurotization of the transferred muscle, and at 6 months, voluntary contraction of the transferred muscle was observed during eyelid closure. These results suggest that a paramedian flap combined with the frontalis muscle and periosteum is a useful option for reconstructing horizontal skin defects involving the orbicularis oculi muscle.

## Introduction

When reconstructing lower eyelid defects, it is necessary to consider not only cosmetic aspects but also the prevention of lower eyelid ectropion and eyelid closure dysfunction. We herein report a case in which the lower eyelid skin and orbicularis oculi muscle were lost due to basal cell carcinoma resection and were successfully reconstructed using a paramedian forehead flap combined with the frontalis muscle and periosteum, resulting in good lower eyelid morphology and function. The patient involved in this study signed an informed consent form after a detailed explanation of the benefits and risks of the procedure was provided.

## Case

**Video 1** Video showing eyebrow elevation and eyelid closure at 6 months postoperatively.


**Fig. 1:**
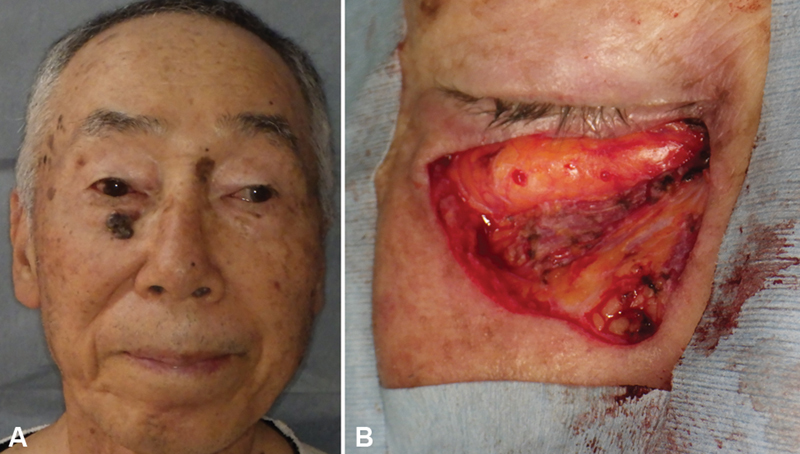
Pre- and post-resection images of the tumor. ( **A** ) Photograph of the tumor prior to resection. ( **B** ) Post-resection image of the tumor, showing the excision of both the tumor and the orbicularis oculi muscle.

The patient was a 75-year-old man, who was aware of a black lesion on his right lower eyelid for approximately 5 years. After the biopsy, the patient was diagnosed with basal cell carcinoma, and resection was performed. The tumor measured 22 mm × 20 mm, the margin was set at 5 mm, and the orbicularis oculi muscle was excised. An artificial dermis made from collagen (Integra®; Integra LifeSciences Corporation, NJ), equipped with drainage holes, was applied to the resected area. This artificial dermis acted as a temporary dressing until pathology results were confirmed, with the dual purpose of minimizing scar contracture of the lower eyelid and optimizing wound bed preparation for subsequent surgery. After pathological examination confirmed negative surgical margins, the patient underwent reconstruction 27 days after the initial surgery ( Fig. 1 and Supplementary Fig. S1 [available in the online version only]). The patient provided informed consent for the use of his medical information, including photography and video, for this case report and publication.

**Fig. 2:**
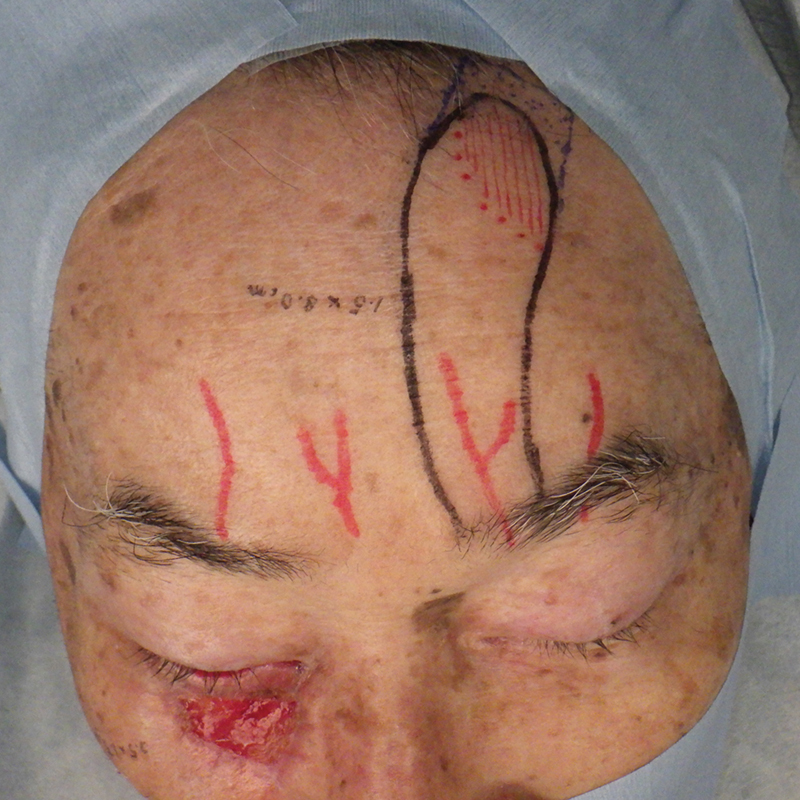
Design of the paramedian forehead flap.

Due to the significant loss of the orbicularis oculi muscle and the expected postoperative development of lower eyelid ectropion, a paramedian forehead flap with frontalis muscle and periosteum was used. The flap, sourced from the contralateral side and pedicled by the supratrochlear artery, was chosen to align the frontalis muscle with the course of the orbicularis oculi muscle after the transfer. The skin island was designed to be 2.5 cm wide and 8.0 cm long, and slightly outward, in line with the running of the frontalis muscle ( Fig. 2 and Supplementary Fig. S2 [available in the online version only]).

**Fig. 3:**
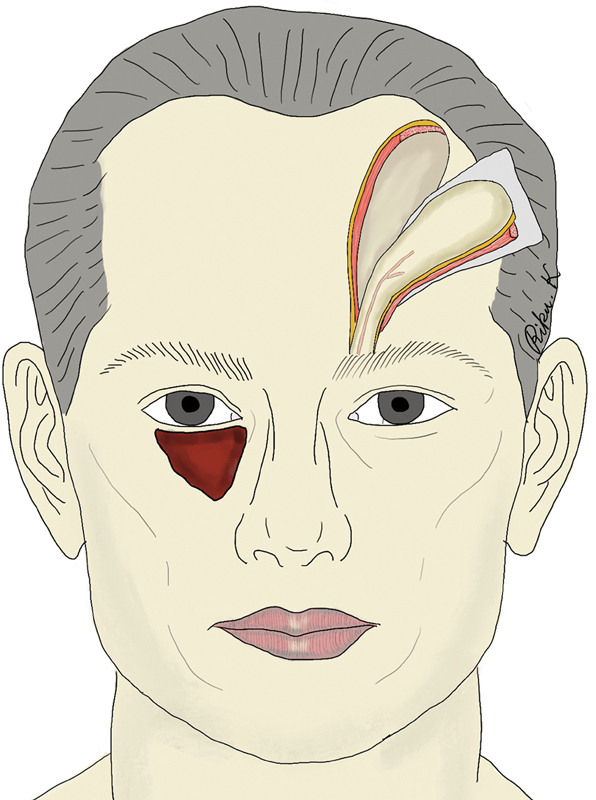
Schematic representation of the flap elevation procedure.

**Fig. 4:**
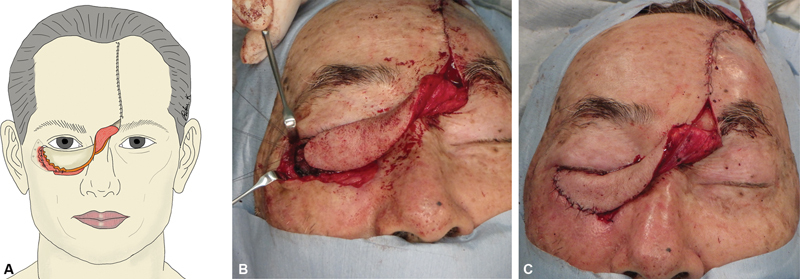
Flap suturing process. ( **A** ) Schematic view. ( **B** ) Intraoperative photograph showing the suturing of the periosteum to the outer orbital periosteum. ( **C** ) Postoperative photograph.

The periosteum was slightly larger than the skin island, and the flap was dissected under the periosteum ( Fig. 3 and Supplementary Figs. S3 and S4 [available in the online version only]). The periosteum was fixed to the outer orbital periosteum with a nonabsorbable thread and used to suspend the lower eyelid. The frontalis muscle was sutured to the residual orbicularis oculi stump to increase the adhesive area. The skin island and surrounding skin were created with a zigzag incision to prevent a trapdoor deformity ( Fig. 4 ).

**Fig. 5:**
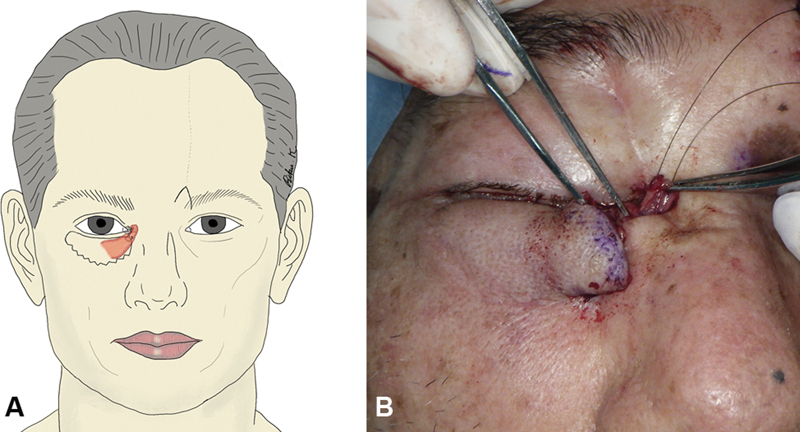
Flap division procedure. ( **A** ) Schematic view. ( **B** ) Intraoperative photograph showing the suturing of the frontalis muscle to the medial palpebral ligament.

Twenty-seven days after flap elevation, flap division was performed, and good bleeding was observed from the stump of the transferred frontalis muscle. A “W” incision was made in the medial canthus, and the stump of the transferred frontalis muscle was sutured to the medial palpebral ligament using a nonabsorbable thread ( Fig. 5 ).

**Fig. 6:**
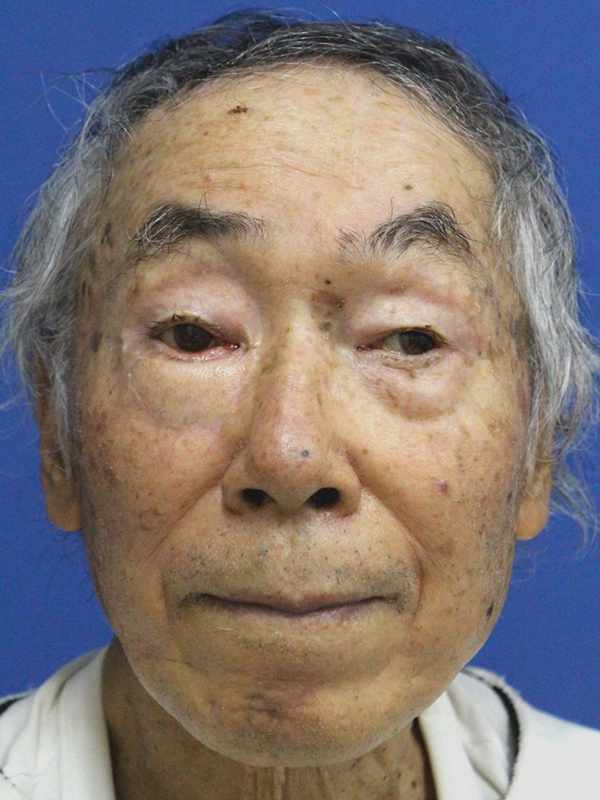
Photograph at 6 months postoperatively.

**Fig. 7:**
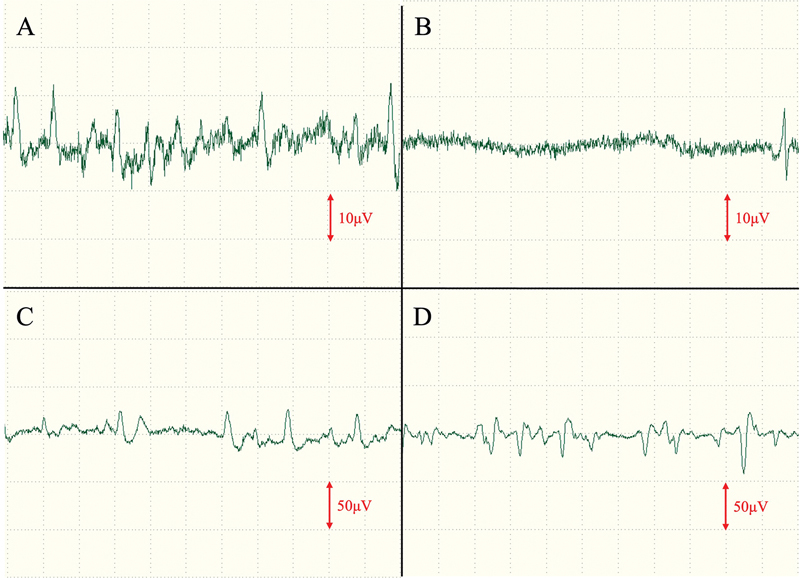
Electromyogram results at 1 year postoperatively. ( **A** ) Normal side, rested. ( **B** ) Skin island, rested. ( **C** ) Normal side, eyelid closed. ( **D** ) Skin island, eyelid closed.

At 6 months postoperatively, the lower eyelid morphology was good, with no evidence of eyelid closure disorders, although a mild degree of ectropion persisted ( Fig. 6 and Supplementary Fig. S5 [available in the online version only]). During eyelid closure, the transplanted frontalis muscle showed voluntary movements ( Video 1 ), and an electromyogram was performed 3 months and 1 year postoperatively to evaluate the neural reinnervation of the transferred frontalis muscle. The results showed a partial resting denervation potential, with voluntary contraction displaying almost the same amplitude as the normal side, suggesting neurotization of the frontalis muscle ( Fig. 7 ). Perceptual evaluation using the Semmes–Weinstein monofilament showed G (3.84) perception at 3 months postoperatively and B (2.36) perception at 1 year postoperatively in the central area of the skin island.

## Discussion

Although local flaps, such as the malar and lateral orbital propeller flaps, have been reported as reconstruction methods for lower eyelid defects, the reach of the flap is limited when the defect is horizontal and extends to the medial canthus. 1 2 3 In addition, it is necessary to prevent postoperative lower eyelid ectropion in patients with orbicularis oculus muscle defects. The surgical procedure reported here has the advantage that not only can the lower eyelid be suspended by a periosteal flap, but the orbicularis oculi muscle defect can also be reconstructed with the muscle. The frontalis muscle is considered to have a dehiscence in the midline of the frontal forehead. By designing a skin island toward the lateral side of the head, it is possible to elevate the skin flap in accordance with the running of the frontalis muscle while simultaneously increasing the length of the skin flap. 4 5 6 The paramedian forehead flap is effective in reconstructing extensive defects of the lower eyelid, especially those with a vertical dimension of approximately 2 cm. 7 8 9 10 The forehead flap used for eyelid reconstruction, while effective, lacked pliability and remained somewhat thick when the eye was closed. Nevertheless, the color and texture of the flap matched that of the lower eyelid, and in this case, the patient did not report any concerns or requests for revision regarding scarring at the frontal donor site. It should also be noted that the defect, in this case, was not full-thickness, and partial loss of the orbicularis oculi muscle may not always necessitate reconstruction of the muscle layer. The decision to proceed with this reconstruction was made after providing a thorough explanation and obtaining informed consent from the patient. Furthermore, this method may not be suitable for all similar defects.

Neurotization by collateral sprouting has been reported in the past. In the present case, muscle-to-muscle neurotization (muscular neurotization) from the surrounding residual orbicularis oculi muscle to the transferred frontalis muscle or nerve-to-muscle neurotization (direct intramuscular neurotization) from a small branch of the cut zygomatic branch of facial nerve to the transferred frontalis muscle might have occurred. 11 Neurotization may prevent ectropion by maintaining muscle tone at rest and reconstructing dynamic function during eyelid closure. Although there have been several reports on lower eyelid reconstruction using a paramedian forehead flap, our case is the first to report the use of a paramedian forehead flap combined with the frontalis muscle and periosteum, as well as the possibility of neurotization of the transferred frontalis muscle.

Despite its disadvantages, such as scarring in the frontal area and being a two-stage procedure, the paramedian forehead flap with the frontalis muscle and periosteum is a useful reconstruction method for vertically extensive lower eyelid tissue defects, including the orbicularis oculi muscle, because it delivers both cosmetic and functional restoration of the lower eyelid. However, the postoperative persistence of mild ectropion in the lower eyelid highlights the need for further refinement of the surgical technique.
