# Supplementary material for: Functional Reconstruction of Lower Eyelid Using Paramedian Forehead Flap Combined with Frontalis Muscle and Periosteum
Source: Arch Plast Surg. 2025 May 15;52(3):132–6. doi: 10.1055/a-2521-2337 (PMC12081096; doi:10.1055/a-2521-2337)
Supplement: Supplementary file 2 — Supplementary Material [file 10-1055-a-2521-2337-s24feb0037cr.pdf]

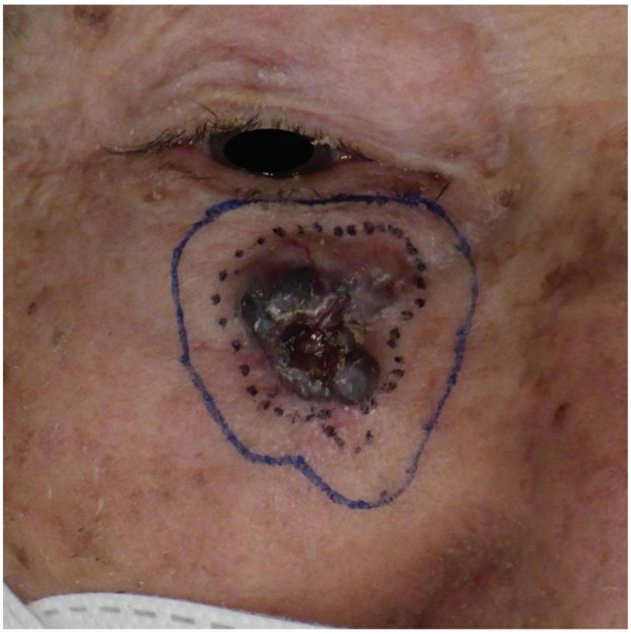

Supplementary Fig. S1 Design of the tumor resection.

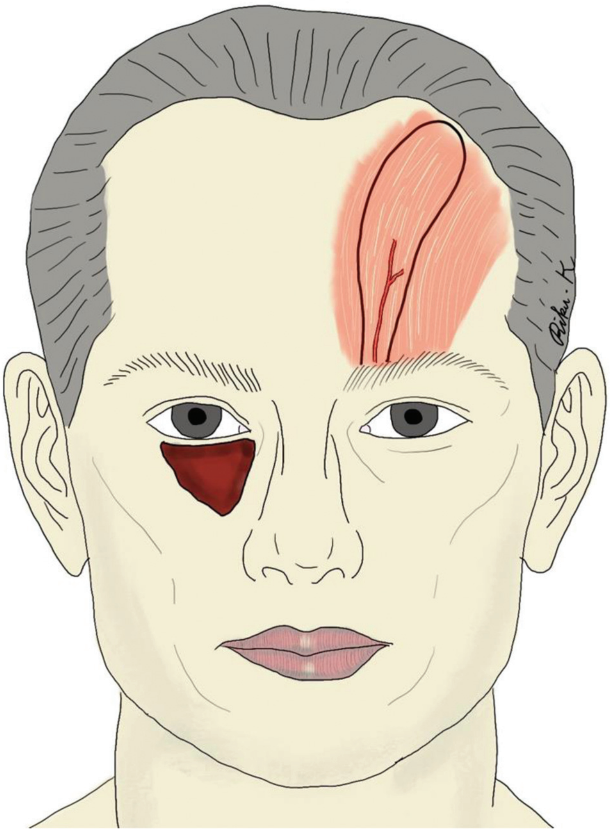

Supplementary Fig. S2 Schematic view of the paramedian forehead flap design.

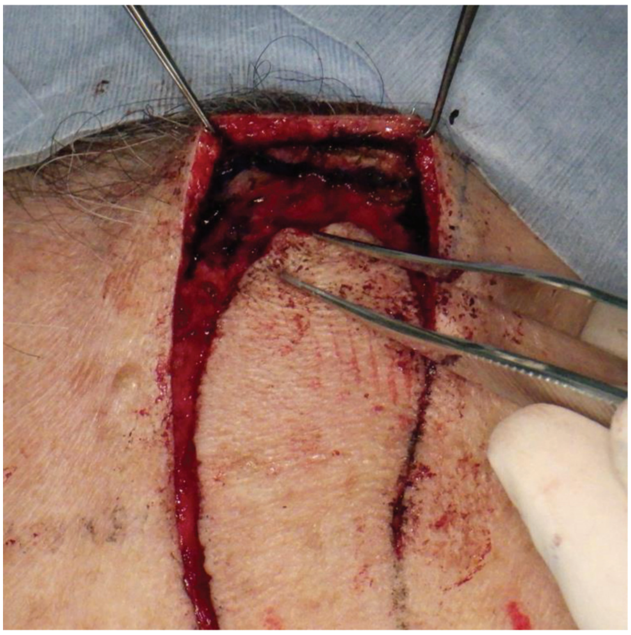

Supplementary Fig. S3 Intraoperative photograph showing flap elevation.

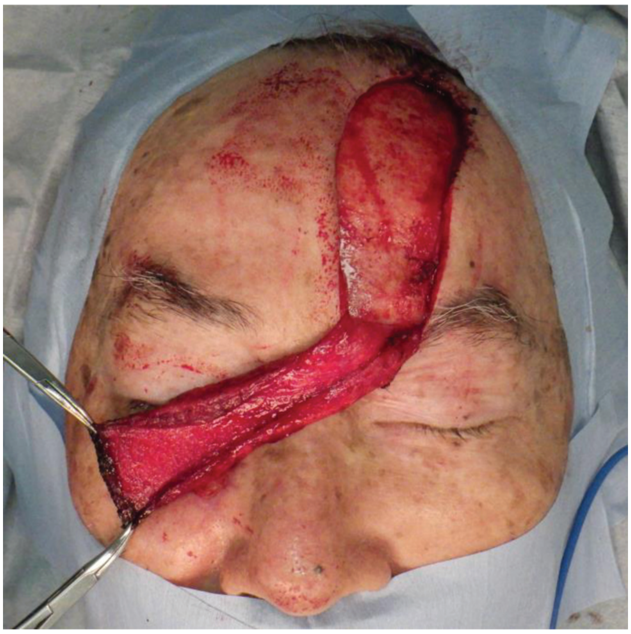

Supplementary Fig. S4 Intraoperative photograph of the elevated flap.

 © 2025. The Author(s).

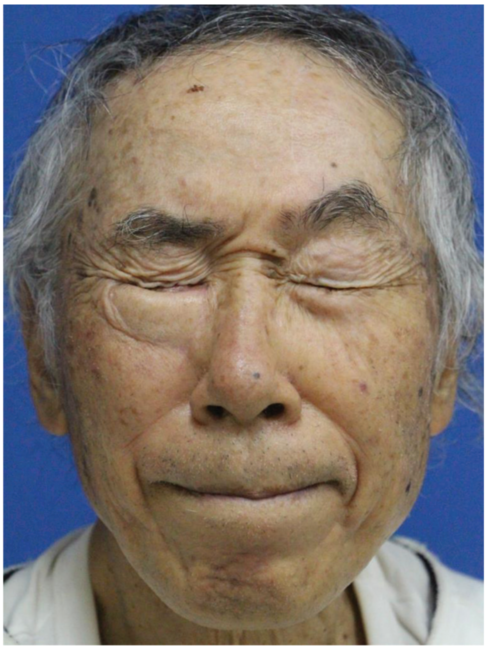

Supplementary Fig. S5 Photograph taken at 6 months postoperatively with the eyelids closed.

 © 2025. The Author(s).
